# Evaluating treatment strategies and machine learning based treatment recommendation system for elderly patients with high grade gliomas

**DOI:** 10.3389/fonc.2025.1597925

**Published:** 2025-08-11

**Authors:** Feiling Xiang, Mengyuan Fu, Xuelian Yang

**Affiliations:** ^1^ Department of Neurosurgery, The First Affiliated Hospital of Chongqing Medical University, Chongqing, China; ^2^ Department of Nursing, The First Affiliated Hospital of Chongqing Medical University, Chongqing, China

**Keywords:** surgery, gross total resection, subtotal resection, adjuvant therapy, treatment recommendation

## Abstract

**Background:**

When selecting treatment strategies, elderly high-grade glioma (eHGG) patients face challenges due to aging, comorbidities, surgical complications, and limited tolerance for intensive treatments. This study aims to evaluate the benefit of treatment strategies and develop a treatment recommendation system for eHGG patients.

**Methods:**

By propensity score matching and survival analysis, we compared the prognosis of treatment strategies, including surgery versus none, adjuvant therapies versus none, and gross total resection (GTR) versus subtotal resection (STR), among patients aged 65 and older with high-grade gliomas. A machine learning model, random survival forest, was developed to provide predictions on prognosis. The machine learning model was then used to create a personalized treatment recommendation system. An independent validation cohort was obtained from the First Affiliated Hospital of Chongqing Medical University to validate the machine learning model and the treatment recommendation system. The time-dependent AUC (tdAUC), C-index, and integrated Brier score (IBS) in the testing sets were obtained.

**Results:**

Compared to the surgery-alone group, patients who received surgery plus adjuvant therapy had significantly better overall survival. Surgery plus adjuvant therapy improved survival compared to adjuvant therapy alone. Additionally, GTR combined with adjuvant therapy showed superior overall survival compared to STR with adjuvant therapy. Subgroup analysis indicated that patients with GBM, tumor size >3 cm, localized stage, white race, Grade IV tumors, and those aged 65–72 had better survival outcomes with GTR and adjuvant therapy. The C-index, tdAUC, and 1-IBS values for the external testing cohort were 0.813, 0.876, and 0.893. We successfully developed a web-based treatment recommendation system at https://gliomas.shinyapps.io/EHGG/. This system allows users to input patient-specific features and obtain individualized treatment recommendations and detailed survival probabilities.

**Conclusion:**

Aggressive treatment, including GTR and adjuvant therapy, can enhance survival outcomes in elderly patients with high-grade gliomas. The machine learning-based personalized treatment recommendation system presents a promising reference tool for treatment decisions.

## 1 Introduction

Gliomas are the most common malignant brain tumors in adults, with an annual incidence of 5.5 per 100,000 individuals (1). High-grade gliomas (grades III and IV, HGG) are the most prevalent and aggressive form of primary brain tumors in adults. HGG is associated with significantly higher mortality rates. For example, the median overall survival of grade IV HGG is only about 15 months (2). The treatment choices for HGG include surgical resection, radiotherapy, chemotherapy, targeted therapy, and immunotherapy (3). Unlike younger patients, elderly HGG (eHGG) patients face challenges to these available treatment choices because of aging, multiple comorbidities, decreased tolerance to chemotherapy, and an increased risk for radiation-induced neurotoxicity (4). Additionally, the risks of surgical complications are higher in those aged 75 and older (5). Thus, whether eHGG patients could benefit from surgical resection, especially GTR, along with adjuvant therapies, is controversial.

Developing precise survival prediction tools is essential to provide personalized treatment strategies for eHGG patients. However, many current studies on HGG (6–8) lack machine learning-based models that are both highly accurate and directly applicable in clinical practice. Besides, existing prognostic models usually focus on survival prediction without offering user-friendly systems for determining appropriate treatment strategies. Bridging this gap is vital for enhancing treatment planning and improving outcomes for eHGG patients.

The first aim of this study is to evaluate the impact of different treatment strategies, including surgery and adjuvant therapy, and compare the benefits of GTR versus STR. This analysis aims to determine whether these treatment approaches significantly benefit eHGG patients. The second aim of this study is to develop a personalized treatment recommendation system using a machine learning-based survival model. By focusing on these aims, our study could contribute to providing personalized treatment strategies.

## 2 Materials and methods

### 2.1 Data resources and patient selection

This study utilized data extracted from the Surveillance, Epidemiology, and End Results (SEER) database. Patient cases diagnosed between January 1, 2000, and December 31, 2021, were obtained from the SEER database using SEER*Stat software. The external and independent testing data used for validation were collected from the First Affiliated Hospital of Chongqing Medical University. In this external hospital, only patients diagnosed with glioma between July 1, 2022, and December 31, 2024, were selected for further analysis. Patients were censored at the date of last known follow-up or the end of the study period if alive, whichever came first. This approach was applied consistently across both cohorts to define overall survival time.

The inclusion criteria were defined as follows: (1) the primary tumor site was the brain; (2) patients were aged 65 years or older; (3) the tumor type was glioma, and the tumor grade was limited to Grade III or IV; (4) the case was the first and primary malignancy; (5) clinical data were available, including marital status, race, histological type, stage, tumor size, surgical intervention, chemotherapy, radiation therapy, overall survival (OS) status, and OS duration; and (6) the diagnosis was confirmed through positive histological findings. These inclusion criteria were consistently applied to the SEER and external testing cohorts. In the SEER and external cohorts, samples with missing data were directly excluded.

### 2.2 Selection of variables

The study variables extracted from the SEER database included demographic variables such as age (65–72 or ≥73), gender (male or female), race (white or minority), and marital status (married or unmarried); clinicopathologic variables including histological type (GBM or non-GBM), tumor size (≤3 cm or >3 cm), stage (localized or advanced), and grade (III or IV); treatment variables such as surgery type (none, STR, or GTR), chemotherapy (no or yes), and radiation therapy (no or yes); and survival data including survival status (alive or dead) and survival duration in months.

According to SEER coding definitions, “None” was defined as no surgery of the primary site or autopsy only. “STR” (subtotal resection) included local tumor destruction, excision of tumor/lesion/mass, or partial resection of the tumor. “GTR” (gross total resection) refers to the complete macroscopic removal of the tumor. These categorizations were based on operative or pathology reports recorded in SEER and may be subject to inter-institutional variability. In the external testing data from The First Affiliated Hospital of Chongqing Medical University, the same definitions for STR and GTR were applied to ensure consistency. The extent of resection was recorded in the surgical record and reported by the operating surgeons.

### 2.3 Propensity score matching analysis

Based on logistic regression, PSM was performed using the nearest-neighbor matching method with a caliper of 0.2 on the propensity scale. PSM was applied to balance various baseline variables between patient groups (9). For example, when matching patients from the surgery alone and surgery with adjuvant therapy groups to investigate the survival effects of adjuvant therapy, the variables for matching included age, gender, marital status, race, histological type, stage, grade, and tumor size. In the PSM analysis, the 1:1 matching ratio was utilized for these groups. Then, Kaplan-Meier analysis was used to assess OS differences between the groups with and without adjuvant therapy. Univariate Cox regression analysis was also performed to explore the effects of adjuvant therapy within different subgroups of the matched data. This matching process was also extended to other analyses, including comparisons between (1) adjuvant therapy alone *vs*. surgery combined with adjuvant therapy and (2) GTR with adjuvant therapy *vs*. STR with adjuvant therapy.

### 2.4 Personalized treatment selection system

This study developed a machine learning-based personalized treatment selection system for older patients with high-grade gliomas. (1) Patients were randomly divided into training and internal testing sets in an 8:2 ratio. A survival prediction model was constructed using the survival random forest algorithm based on clinical variables and treatment-related variables. The training process employed 5-fold cross-validation in the training set. The model was trained using different parameters of ntree values (100, 200, 500, 1000) and mtry values (1, 2, 4, 6). The parameter combination yielding the highest time-dependent area under the curve (tdAUC) during cross-validation was selected for constructing the final model. (2) The model’s performance was validated on the internal and external testing sets using the C-index, tdAUC, and integrated Brier score (IBS). The validation process was repeated 20 times to enhance robustness, with 50% of the testing sets randomly selected as validation subsets in each iteration. (3) To assess the model’s treatment recommendation ability, the survival outcomes of patients under all 12 treatment options in the internal and external testing sets were predicted using the trained model. The treatment associated with the highest predicted survival is the model’s recommendation. Patients were categorized into consistent (Cons) and inconsistent (Inco) groups based on whether their actual treatment aligned with the model’s recommendation. The survival curves of the Cons and Inco groups were compared, and the corresponding survival curves were plotted.

Then, variable importance was calculated using the permutation importance method with tdAUC as the evaluation metric. The process involved first computing the original tdAUC and then iteratively permuting each variable in the testing data to disrupt its relationship with the outcome. The decrease in mean tdAUC following permutation was used to quantify the importance of the variable. The importance scores were normalized using Min-Max scaling to provide relative importance values. To enable direct comparison with published nomograms for survival prediction, typically based on classical Cox models, we selected three representative nomograms from the literature. These three nomogram models were named as nomo1 (10), nomo2 (11), and nomo3 (12). For each, we reproduced the model according to the variables in the original publication and computed tdAUC values using our testing dataset.

### 2.5 Development of the online system

We developed a user-friendly treatment recommendation interface that allows users to input relevant features and obtain model-generated treatment recommendations by simply clicking the “Recommend” button. The interface displays individualized survival probability curves and associated values for various treatment options. Additionally, the system calculates the mean survival probability differences between treatment options, including the baseline option of no surgery, no chemotherapy, or no radiation therapy. These mean survival probability differences, interpreted as the survival benefit, are also presented within the online system. The treatment option with the highest survival benefit is the most recommended choice.

### 2.6 Statistical analysis

Statistical analyses were performed using R version 4.3.2. Categorical variables were presented as numbers and percentages (%). Cox proportional hazards (CPH) models were used to calculate hazard ratios (HR), and the log-rank test was applied to compare Kaplan–Meier (KM) survival curves.

## 3 Results

### 3.1 Percentages of treatment strategies

**Figure 1:**
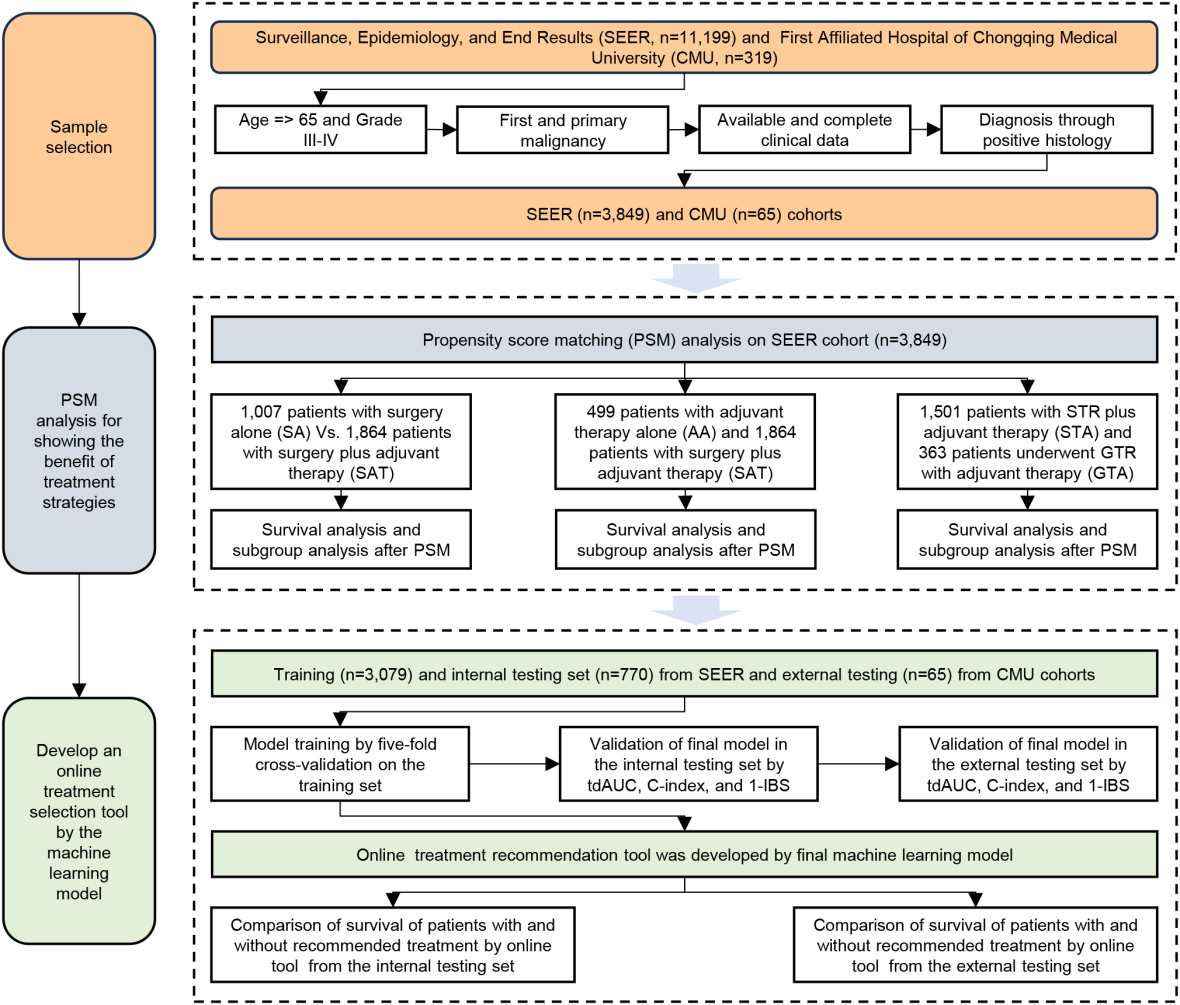
Study flowchart of this study.

The workflow of this study is illustrated in **Figure 1**. Based on our inclusion criteria, 3,849 patients with eHGG from the SEER database were identified. The frequencies and percentages of treatment strategies among these 3,849 patients are summarized in **Table 1**. Subtotal resection (STR) combined with chemotherapy and radiation therapy emerged as the most common treatment strategy (39.0% of cases). This was followed by non-surgical treatment with chemotherapy and radiation (13.0% of cases) and STR without chemotherapy or radiation therapy (11.4% of cases). Gross total resection (GTR) with chemotherapy and radiation therapy was employed in 9.4% of cases, while STR without chemotherapy but with radiation therapy accounted for 7.7%. The median survival time for patients in the SEER cohort was 0.5 years.

**Table 1:** Frequencies and percentages of treatment strategies among elderly patients with high-grade glioma from SEER database.

Surg	Chem	Rad	Comb	Freq	Perc
STR	Yes	Yes	S|Y|Y	1,501	39.0
None	Yes	Yes	N|Y|Y	499	13.0
STR	No	No	S|N|N	438	11.4
GTR	Yes	Yes	G|Y|Y	363	9.4
STR	No	Yes	S|N|Y	295	7.7
None	No	No	N|N|N	285	7.4
None	No	Yes	N|N|Y	165	4.3
GTR	No	No	G|N|N	101	2.6
GTR	No	Yes	G|N|Y	78	2.0
STR	Yes	No	S|Y|N	77	2.0
None	Yes	No	N|Y|N	29	0.8
GTR	Yes	No	G|Y|N	18	0.5

The treatment strategies are categorized based on surgery type (subtotal resection [STR], gross total resection [GTR], or none), chemotherapy (Chem), and radiation therapy (Rad). The combination of treatment modalities is detailed in the “Comb” column, with the corresponding frequency (Freq) and percentage (Perc) of each strategy provided.

Based on the same inclusion criteria, 65 patients with eHGG were identified from 319 patients in the external testing cohort from The First Affiliated Hospital of Chongqing Medical University. The distribution of treatment strategies among these patients is summarized in **Table 2**. The most commonly adopted treatment approach was GTR combined with both chemotherapy and radiation therapy, accounting for 35.4% of cases. This was followed by GTR without chemotherapy or radiation therapy (29.2%) and GTR with chemotherapy but without radiation therapy (24.6%). The median survival time for patients in the external testing cohort was 0.8 years.

**Table 2:** Frequencies and percentages of treatment strategies among elderly patients with high-grade glioma from the external testing set.

Surg	Chem	Rad	Comb	Freq	Perc
GTR	Yes	Yes	G|Y|Y	23	35.4
GTR	No	No	G|N|N	19	29.2
GTR	Yes	No	G|Y|N	16	24.6
STR	No	No	S|N|N	3	4.6
GTR	No	Yes	G|N|Y	2	3.1
STR	Yes	Yes	S|Y|Y	2	3.1

### 3.2 Surgery alone *vs*. surgery with adjuvant therapy

**Figure 2:**
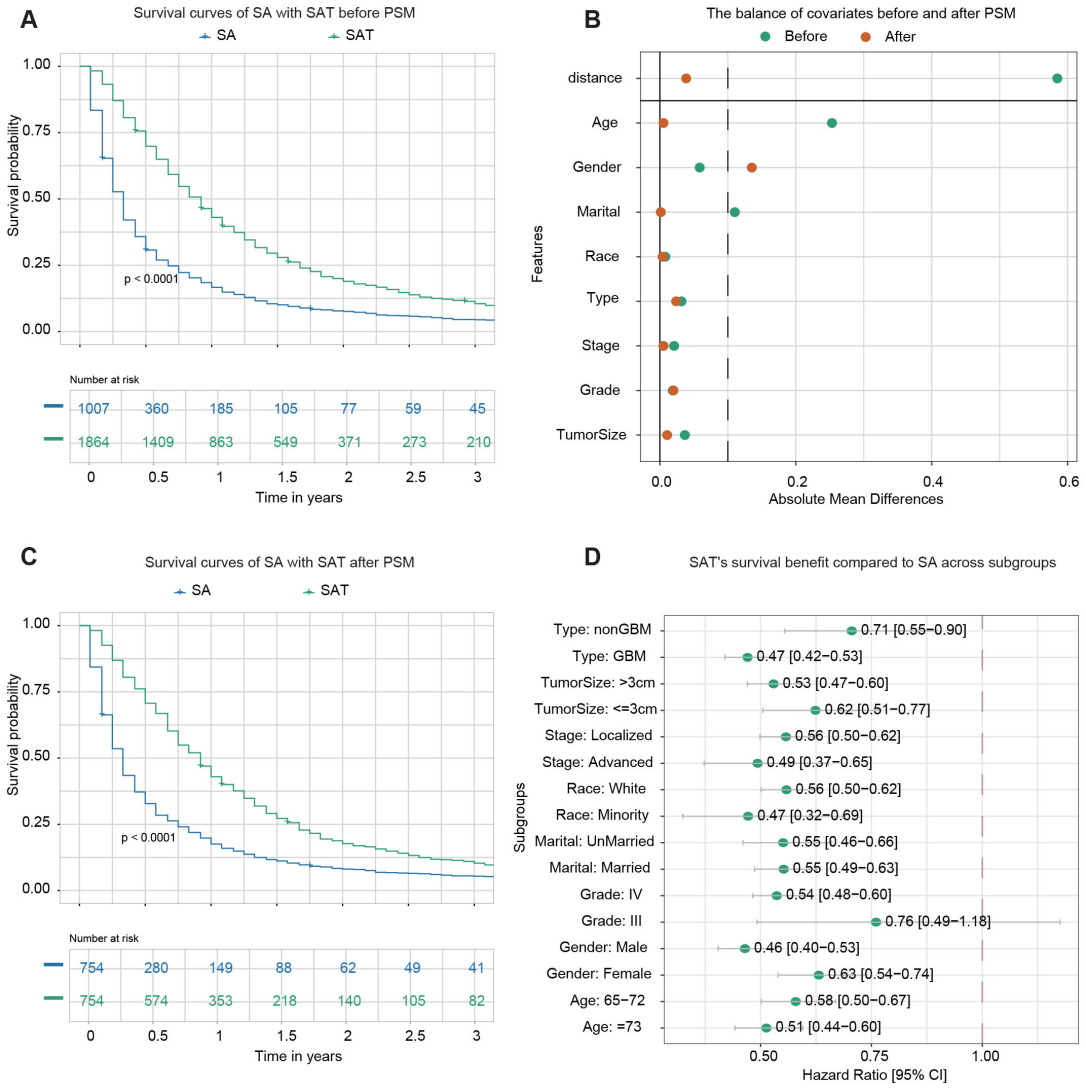
Survival analysis of surgery alone (SA) *vs*. surgery plus adjuvant therapy (SAT). **(A)** Kaplan–Meier survival curve comparing SA with SAT before PSM. **(B)** The balance of covariates before and after PSM indicates improved stability in the distribution of key variables after matching. **(C)** Kaplan–Meier survival curve comparing SA with SAT after PSM. **(D)** The forest plot from univariate Cox regression analysis shows SAT’s survival benefit compared to SA across subgroups. For instance, among samples from the non-GBM subgroup, the forest plot highlights that SAT significantly improves survival outcomes compared to SA.

In this section, we investigated the impact of adjuvant therapy on the survival outcomes. Adjuvant therapy was defined as the combination of chemotherapy and radiation therapy administered following surgery. 1,007 patients underwent SA (either STR or GTR), while 1,864 patients received SAT. The demographic and clinicopathological characteristics of the two groups before PSM are summarized in **Supplementary Table S1**. Before PSM, Kaplan-Meier analysis revealed a statistically significant difference (p-value<0.0001) in OS between the two groups (**Figure 2A**). The 1-year OS rates were 16.6% (95% CI: 14.5–19.1%) for patients who underwent SA and 43.0% (95% CI: 40.8–45.3%) for those who received SAT.

PSM was performed to address potential confounding factors. A total of 754 matched pairs of patients who underwent SA or SAT were identified in a 1:1 ratio. After matching, the standard mean difference of all parameters indicated that significant differences in baseline characteristics were reduced (**Figure 2B**). The demographic and clinicopathological characteristics of the two groups after PSM are presented in **Supplementary Table S2**. No significant difference was found among variables except gender. Following PSM, Kaplan-Meier analysis still demonstrated a statistically significant difference in OS between patients who received SAT and those who underwent SA (p-value<0.0001) (**Figure 2C**). The 1-year OS rates were 17.5% (95% CI: 15.0–20.4%) for the SA group and 42.9% (95% CI: 39.5–46.6%) for the group receiving SAT.

Next, a subgroup analysis was conducted to examine the effect of adjuvant therapy on survival outcomes within each subgroup. Univariate Cox regression analysis revealed that adjuvant therapy was associated with improved survival in most subgroups, except for patients identified as belonging to Grade III subgroups (**Figure 2D**).

### 3.3 Adjuvant therapy alone *vs*. surgery plus adjuvant therapy

**Figure 3:**
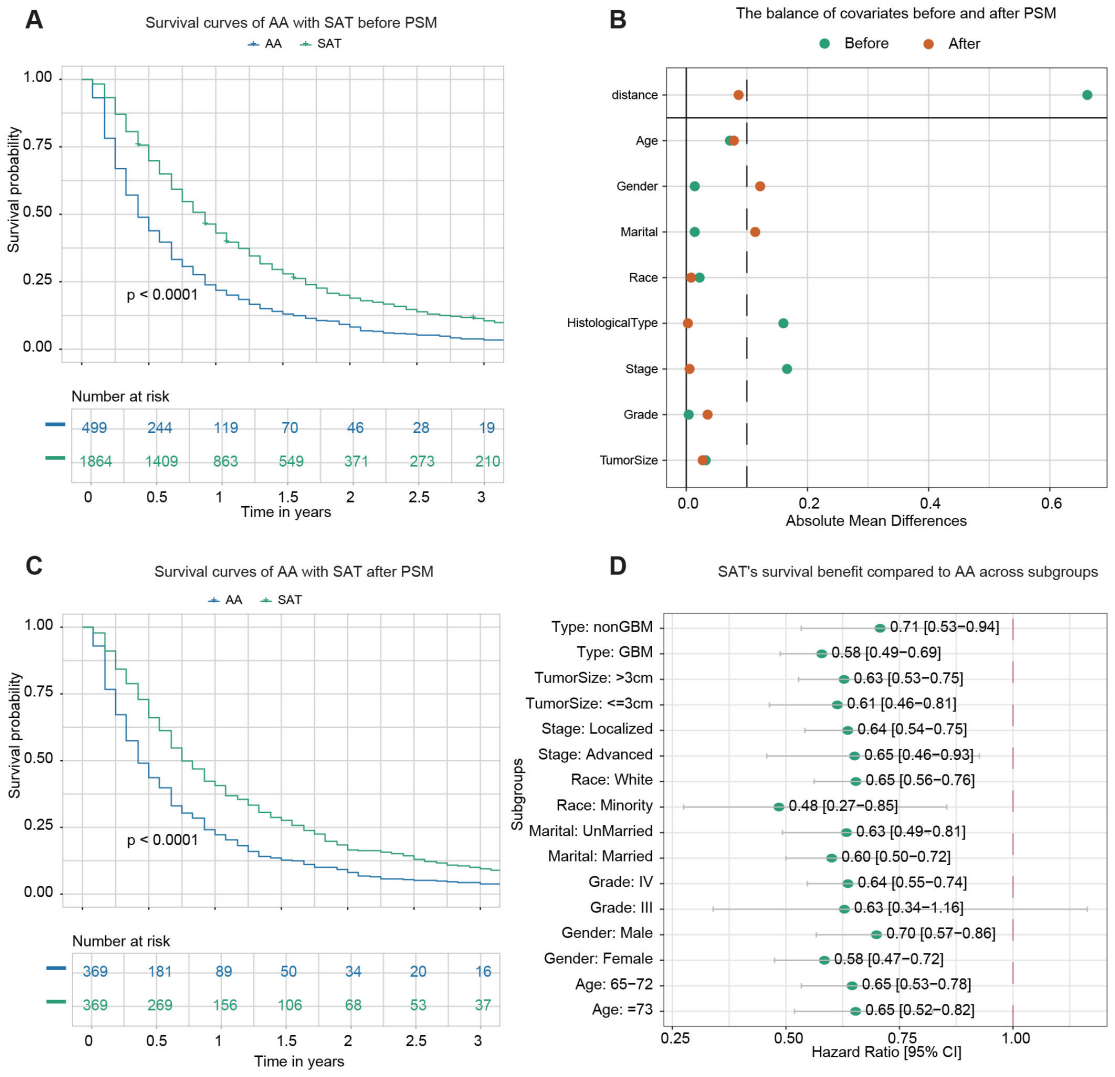
Survival analysis of adjuvant therapy alone (AA) *vs*. surgery plus adjuvant therapy (SAT). **(A)** Kaplan–Meier survival curve comparing AA with SAT before PSM. **(B)** The balance of covariates before and after PSM. **(C)** Kaplan–Meier survival curve comparing AA with SAT after PSM. **(D)** The forest plot from univariate Cox regression analysis shows SAT’s survival benefit compared to AA across subgroups.

499 patients underwent adjuvant therapy alone, while 1,864 patients received adjuvant therapy combined with surgery, either STR or GTR. The demographic and clinicopathological data of the two groups before PSM are summarized in **Supplementary Table S3**. Before PSM, Kaplan-Meier analysis revealed a statistically significant difference (p < 0.0001) in OS between the two groups (**Figure 3A**). The 1-year OS rates were 21.8% (95% CI: 18.5–25.7%) for patients receiving adjuvant therapy alone and 43.0% (95% CI: 40.8–45.3%) for those undergoing surgery with adjuvant therapy.

After PSM, 369 pairs of patients from the AA and SAT groups were matched in a 1:1 ratio. After matching, the standard mean difference of parameters indicated that most differences in baseline characteristics were reduced between the two cohorts (**Figure 3B**). The demographic and clinicopathological data of the two groups after PSM are presented in **Supplementary Table S4**. No significant difference was found among variables except age, gender, and marital status. Kaplan-Meier analysis post-PSM demonstrated a statistically significant difference in OS between the two groups (p < 0.0001) (**Figure 3C**). The 1-year OS rates were 22.2% (95% CI: 18.3–26.8%) for the AA group and 40.6% (95% CI: 35.9–45.9%) for the SAT group.

Subgroup analysis was then conducted to evaluate the effect of surgery on survival outcomes in various subgroups. Univariate Cox regression analysis revealed that adjuvant therapy was associated with improved survival in most subgroups, except for patients identified as belonging to Grade III subgroups (**Figure 3D**).

### 3.4 STR and adjuvant therapy *vs*. GTR and adjuvant therapy

**Figure 4:**
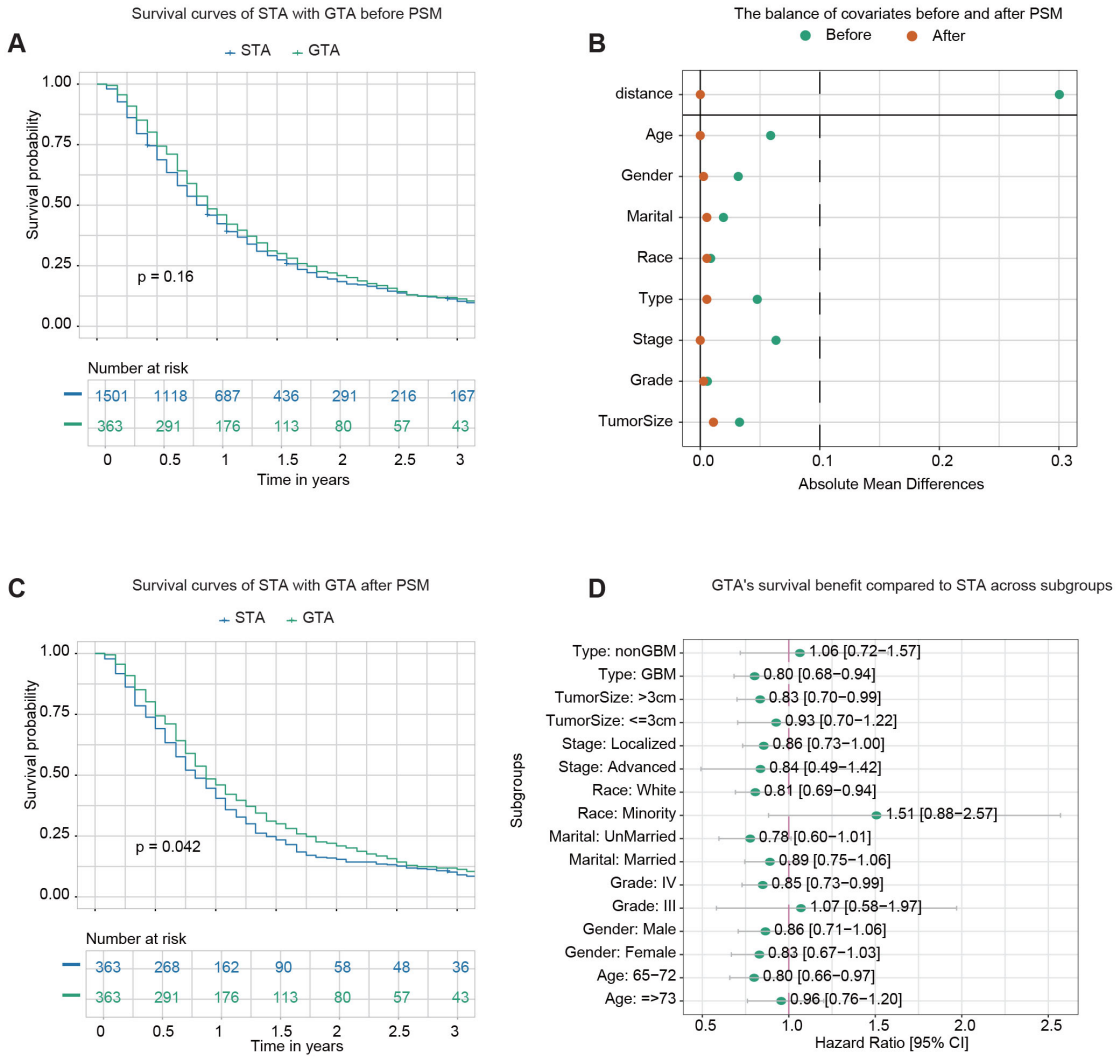
Survival analysis of GTR plus adjuvant therapy (GTA) *vs*. STR plus adjuvant therapy (STA). **(A)** Kaplan–Meier survival curve comparing STA and GTA before propensity score matching (PSM). **(B)** The balance of covariates before and after PSM indicates improved stability in the distribution of critical variables after matching. **(C)** Kaplan–Meier survival curve comparing STA and GTA after PSM. **(D)** The forest plot from univariate Cox regression analysis demonstrates GTA’s survival benefit compared to STA across subgroups.

1,501 patients received STR with adjuvant therapy (STA), and 363 underwent GTR with adjuvant therapy (GTA). The demographic and clinicopathological characteristics of the STA and GTA groups before PSM are summarized in **Supplementary Table S5**. Before PSM, Kaplan–Meier analysis revealed no statistically significant (p-value=0.16) difference in OS between the two groups (**Figure 4A**). The 1-year OS rates were 42.3% (95% CI: 39.9–44.9%) for patients receiving STA and 46.0% (95% CI: 41.1–51.4%) for those undergoing GTA.

After PSM, 363 pairs of patients from the STA and GTA groups were matched in a 1:1 ratio. After matching, the standard mean difference of all parameters indicated that the variables’ differences were reduced (**Figure 4B**). The demographic and clinicopathological characteristics of the two groups after PSM are presented in **Supplementary Table S6**. No significant differences in baseline characteristics between the two cohorts were found. Kaplan–Meier analysis after PSM demonstrated a statistically significant difference in OS between the two groups (p-value=0.042) (**Figure 4C**). The 1-year OS rates were 40.4% (95% CI: 35.7–45.8%) for the STA group and 46.0% (95% CI: 41.1–51.4%) for the GTA group.

Subgroup analysis was conducted to evaluate the impact of more advanced surgery (GTR) on survival outcomes across various subgroups. Univariate Cox regression analysis demonstrated that GTA improved survival in most subgroups, including GBM, tumor size >3 cm, localized stage, white race, Grade IV tumors, and patients aged 65–72 (**Figure 4D**).

### 3.5 Development and validation of the prognostic models

**Figure 5:**
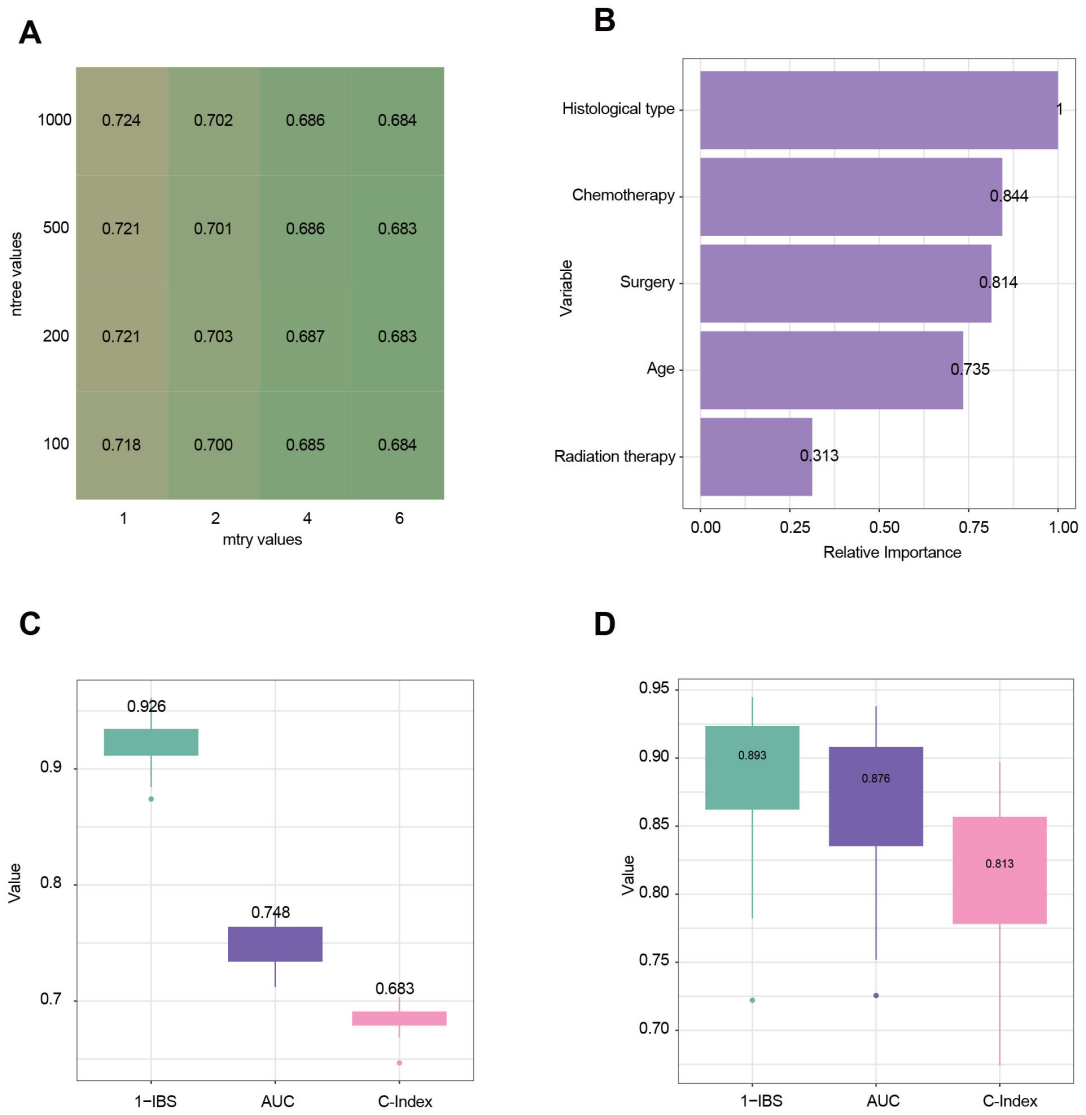
Evaluation and analysis of model performance and variable importance. **(A)** Heatmap illustrating the tuning of hyperparameters for the random survival forest (RSF) model. Rows represent the number of trees (ntree), and columns represent the number of randomly selected variables at each split (mtry). The values in the cells represent the performance metric of the time-dependent Area Under the Curve (tdAUC). **(B)** Variable importance plot derived from the RSF model. The relative importance of each variable is measured. **(C)** Model performance in the internal testing cohort using three evaluation metrics: 1-IBS (Integrated Brier Score), tdAUC, and C-Index (Concordance Index). Higher values indicate better predictive performance. **(D)** Model performance in the external testing cohort.

Using the survival random forest algorithm, a model was developed to predict OS in eHGG patients. 3,849 eHGG patients were randomly divided into training (n=3,079) and internal testing sets (n=770) at the 8:2 ratio. Following hyperparameter tuning via five-fold cross-validation, the optimal parameters were determined to be ntree = 1000 and mtry = 1, which were used for final model training (**Figure 5A**). The relative importance of clinical variables in the final model is illustrated in **Figure 5B**. Histological type and chemotherapy are the most critical variables for the model. The performance of the final model was evaluated using the internal and external testing sets. The C-index, tdAUC, and 1-IBS values for the internal testing cohort were 0.683 (95% CI: 0.677–0.688), 0.748 (95% CI: 0.740–0.756), 0.926 (95% CI: 0.916–0.935) (**Figure 5C**). The C-index, tdAUC, and 1-IBS values for the external testing cohort were 0.813 (95% CI: 0.786–0.840), 0.876 (95% CI: 0.849–0.902), 0.893 (95% CI: 0.868–0.917) (**Figure 5D**). To highlight the advantages of our machine learning model, we also calculated the tdAUC values for three published nomograms as a comparison. In the external validation cohort, the tdAUC values for these nomograms were 0.776, 0.766, and 0.772, respectively (**Supplementary Figure S1**).

The univariate Cox analysis revealed that patients aged 65–72, with localized tumors, non-GBM types, had significantly better survival outcomes, while unmarried patients and those with tumorSize >3 cm had worse outcomes (**Table 3**). Chemotherapy, radiation, and surgery (STR or GTR) were all significantly associated with improved survival, whereas gender, race, and tumor grade showed no significant effects. Besides, in the univariate Cox regression analysis, compared with STR, GTR was associated with a decreased risk of events (HR = 0.907, 95% CI: 0.826–0.996, p = 0.04).

**Table 3:** The univariate Cox analysis of variables in the training set.

Variable	Level	Hazard ratio	Lower 95% CI	Upper 95% CI	P-value
Age	=>73 (Reference)				
	65-72	0.658	0.617	0.702	<0.001
Gender	Female (Reference)				
	Male	1.032	0.968	1.100	0.335
Marital	Married (Reference)				
	UnMarried	1.253	1.171	1.340	<0.001
Race	Minority (Reference)				
	White	1.098	0.979	1.212	0.111
Type	GBM (Reference)				
	Non-GBM	0.747	0.693	0.806	<0.001
Stage	Advanced (Reference)				
	Localized	0.673	0.620	0.731	<0.001
Grade	III (Reference)				
	IV	0.927	0.816	1.052	0.241
Tumor Size	<=3cm (Reference)				
	>3cm	1.090	1.014	1.171	0.019
Surgery	None (Reference)				
	STR	0.559	0.518	0.603	<0.001
	GTR	0.507	0.456	0.563	<0.001
Chemotherapy	No (Reference)				
	Yes	0.463	0.433	0.496	<0.001
Radiation	No (Reference)				
	Yes	0.427	0.396	0.461	<0.001

### 3.6 Clinical application of the personalized treatment selection system

**Figure 6:**
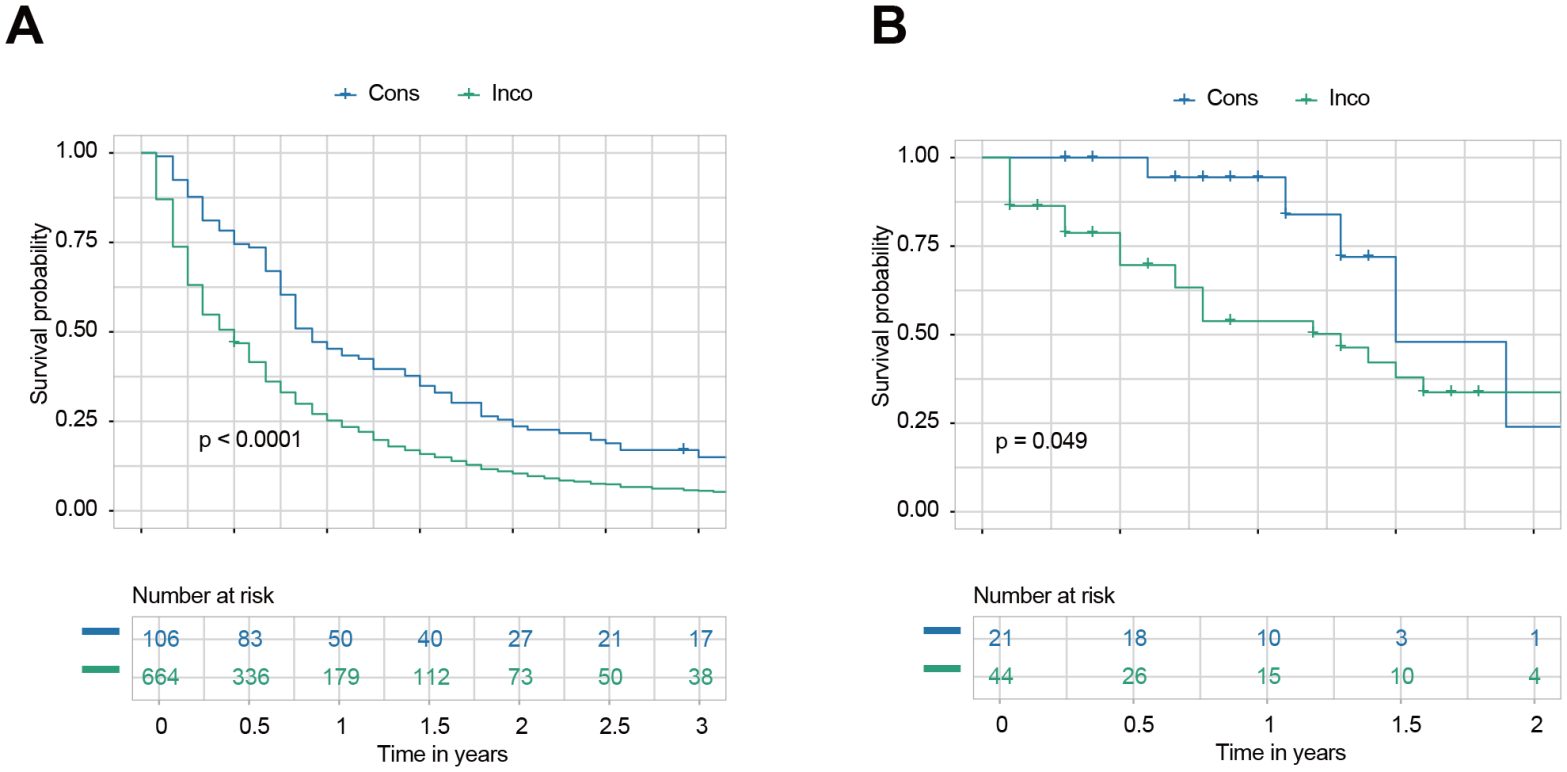
Survival outcomes based on consistency with model recommendations. **(A)** Kaplan-Meier survival curves for all patients in the internal testing set were categorized into consistent (Cons) and inconsistent (Inco) groups based on whether their actual treatment aligned with the model’s recommendation. **(B)** Kaplan-Meier survival curves for all patients in the external testing set were categorized into Cons and Inco groups based on whether their actual treatment aligned with the model’s recommendation.

We calculated the survival probabilities of each patient in the internal and external testing sets across all treatment choices. Treatment associated with the highest predicted survival was selected as the model’s recommendation. Patients were categorized into two groups based on whether their actual treatment aligned with the model’s recommendation: consistent (Cons) and inconsistent (Inco). In the internal testing set, the Cons group showed significantly better survival than the Inco group for all patients (**Figure 6A**). The 1.5-year OS rates were 34.9% (95% CI: 26.9–45.2%) for the Cons group and 15.8% (95% CI: 13.3–18.9%) for the Inco group. Similarly, the Cons group showed significantly better survival in the external testing set than the Inco group for all patients (**Figure 6B**). Within the 1.5 year of post-treatment, the survival of the Cons group is specifically better than that of the Inco group since the OS rates were 47.9% (95% CI: 19.6–100%) for the Cons group and 37.9% (95% CI: 23.8–60.3%) for the Inco group.

### 3.7 Web-based personalized treatment selection system

**Figure 7:**
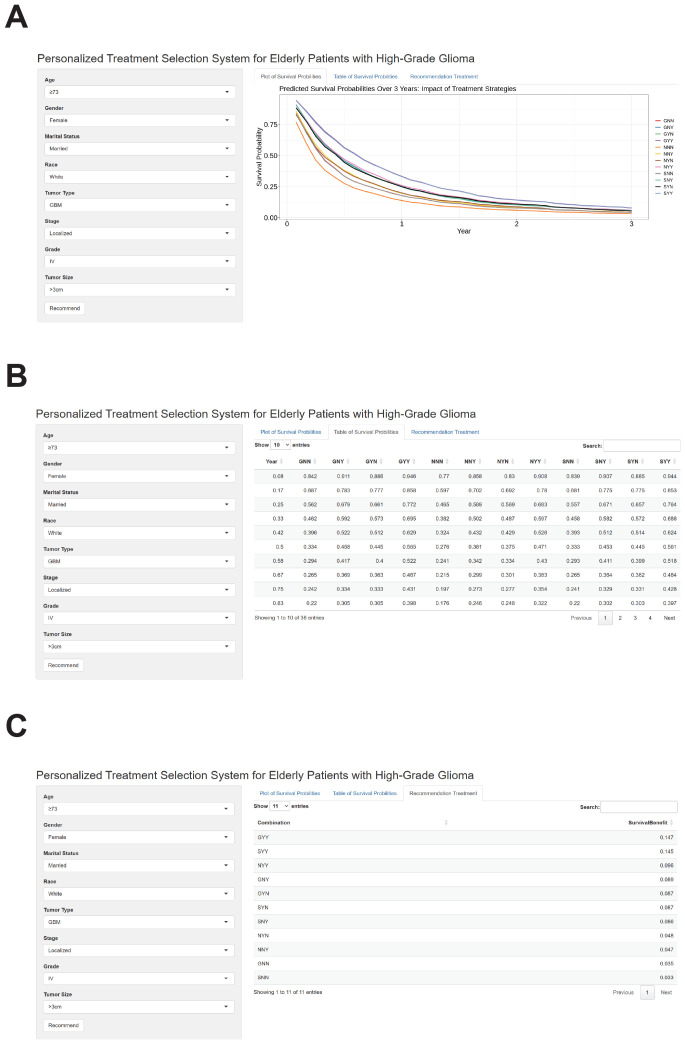
Outputs of the web-based personalized treatment selection system for elderly patients with high-grade glioma. **(A)** The web-based treatment recommendation interface allows users to input patient-specific features like age, gender, marital status, race, histological type, grade, stage, and tumor size. After clicking the “Recommend” button, the system generates individualized survival probability curves for various treatment options. These treatment strategies are represented by three letters, where the first letter indicates the type of surgery (G: gross total resection, S: subtotal resection, N: none), the second letter indicates chemotherapy (N: none, Y: received), and the third letter indicates radiotherapy (N: none, Y: received). **(B)** Detailed numerical values corresponding to the individualized survival probability curves are displayed in a tabular format. These values allow for precise comparisons of survival probabilities across different treatment strategies. **(C)** Calculated survival benefits are presented as mean survival probability differences for each treatment option compared to the baseline (no surgery, no chemotherapy, or no radiation therapy). The treatment option associated with the highest survival benefit is highlighted as the most recommended choice.

We successfully developed a user-friendly, web-based treatment recommendation system, accessible at https://gliomas.shinyapps.io/EHGG/. This system integrates predictive modeling into an interactive interface that allows users to input patient-specific features, including age, gender, marital status, race, histological type, stage, grade, and tumor size. The system outputs three key results by clicking the “Recommend” button. First, it generates survival curves of individualized survival probabilities for various treatment options (**Figure 7A**). Second, it provides detailed numerical values corresponding to these survival probability curves (**Figure 7B**). Third, the system calculates survival benefits by comparing the mean survival probabilities of each treatment option to the baseline option (no surgery, no chemotherapy, no radiation therapy). The treatment option associated with the highest survival benefit is automatically highlighted as the most recommended choice (**Figure 7C**).

## 4 Discussion

Our study aimed to evaluate the impact of various treatment strategies on eHGG patients and to develop a personalized treatment recommendation system using a machine learning-based survival model. The results of this study provide several key insights that can be a potential reference for older patients with high-grade gliomas in clinical decision-making.

At present, there is ongoing controversy about adopting subtotal resection (STR) or gross total resection (GTR) for eHGG patients (13). One of the primary findings of our study is that treatment strategies involving surgical resection, particularly GTR combined with adjuvant therapy, provide significant survival benefits to eHGG patients compared to other treatment modalities. Specifically, GTR combined with adjuvant therapy was associated with significantly improved OS compared to STR combined with adjuvant therapy. This result is particularly crucial given that only 9.4% of patients in our study received GTR with adjuvant therapies, while 39.0% received STR with adjuvant therapies. Our findings also support previous meta-analyses and review studies, which have demonstrated that GTR is associated with a significant improvement in OS compared to STR (13–16). Additionally, GTR has been shown to benefit glioma patients by improving seizure control and reducing the incidence of malignant transformation (13). These results underscore the importance of using GTR in elderly patients as much as possible when the surgical risk is acceptable. Importantly, our subgroup analysis further revealed that patients with GBM, tumor size greater than 3 cm, localized stage, white race, Grade IV tumors, and those aged 65–72 had better survival outcomes with GTR, indicating that they are more suitable candidates for GTR.

The use of adjuvant therapy in high-grade gliomas remains controversial, particularly given the disappointing results from some clinical trials involving adjuvant chemotherapy regimens (17). The blood-brain barrier, which plays a crucial role in maintaining the stability of brain tissue under normal physiological conditions, also poses a significant obstacle to achieving effective chemotherapy concentrations at the tumor site (18). However, other studies have demonstrated that postsurgical adjuvant treatment can improve survival outcomes compared to surgery alone (19). The question of whether chemotherapy should be used in elderly high-grade glioma patients is still debated. Our study demonstrates that for patients aged 65 and older, the use of adjuvant therapies in combination with surgery significantly improves survival compared to surgery alone. This finding suggests that adjuvant therapies, including chemotherapy and radiation, can offer substantial survival benefits for elderly patients with high-grade gliomas. However, our results also indicate that patients with grade III tumors did not experience a statistically significant survival benefit from adjuvant therapy. This lack of significance may be attributed to the limited sample size and statistical power in the grade III cohort. It may also reflect biological heterogeneity, as well as the limitations of available clinical and molecular data.

In recent years, many studies have constructed models for predicting survival in gliomas. For example, a study on adult glioma patients developed nomograms with a C-index of 0.738 in the validation set (10). Another study on GBM reported a C-index of 0.724 (95% CI: 0.713–0.735) in the validation cohort for prognosis prediction (11). Additionally, a study provided a nomogram with a C-index of 0.734 (95% CI: 0.718–0.750) (12). These studies commonly employ nomograms for survival prediction because they are simple and easy-to-understand tools. Unlike these traditional models, we utilized a machine learning approach for survival prediction. The C-index, tdAUC, and 1-IBS values for our machine learning model in the external testing cohort were 0.813, 0.876, and 0.893 (**Figure 5D**). To highlight the advantages of our machine learning model, we also calculated the tdAUC values for three published nomograms as a comparison. In the external validation cohort, the tdAUC values for these nomograms were 0.776, 0.766, and 0.772, respectively (**Supplementary Figure S1**). Although using a machine learning model increases model complexity, we believe that the improvement in tdAUC from 0.766–0.772 to 0.875 is substantial and justifies this increased complexity. Another key strength of our study is developing a personalized treatment recommendation tool based on the survival prediction model. Pure survival prediction alone has limited clinical utility if it does not lead to actionable insights for patient management. Our model goes beyond survival prediction by providing optimal treatment recommendations to each patient. Our results showed that patients whose actual treatments aligned with the model’s recommendations experienced significantly better survival outcomes than those whose treatments differed from the model’s suggestions. This finding indicates that our approach is valuable tool for refining treatment strategies and improving personalized care for elderly glioma patients.

Our findings have important clinical implications for managing eHGG patients. First, the findings underscore the importance of maximizing surgical intervention when feasible, since GTR could improve survival outcomes. Second, the findings emphasize the benefit of adjuvant therapies in enhancing survival, which should be considered even in elderly patients who may face challenges tolerating aggressive treatments. Lastly, developing the machine learning-based personalized treatment recommendation system provides a precise and convenient tool for selecting treatments.

However, several limitations should be acknowledged. First, the SEER database lacks important prognostic variables such as performance status (e.g., Karnofsky Performance Status), comorbidity indices (e.g., Charlson Comorbidity Index), tumor location, underlying diseases (e.g., coronary heart disease), smoking and alcohol consumption status, and tumor molecular markers (e.g., IDH mutation status, MGMT promoter methylation). The absence of these factors may introduce residual confounding and indication bias, as patients with better performance status and fewer comorbidities are more likely to receive aggressive treatment. The absence of these factors can also potentially lead to an overestimation of the survival benefit associated with more aggressive treatment strategies like surgery with adjuvant therapy.

Moreover, the lack of detailed treatment protocols for chemotherapy and radiotherapy represents another limitation. There is also potential for inter-operator and inter-institutional variability in reporting resection rates. Practical barriers and input feasibility issues also exist, as some clinical variables are frequently missing for many patients. Furthermore, perioperative risks, such as morbidity in elderly patients, were not included in the model, despite being important considerations in real-world clinical practice. Finally, the relatively small sample size of the external validation cohort may affect the generalizability of our findings. To address this, we plan to collect a larger, multi-institutional dataset in future studies to further strengthen the robustness and reliability of our analysis.

## 5 Conclusion

In conclusion, our study highlights the significant survival benefits of aggressive treatment strategies (GTR and adjuvant therapies) for elderly patients with high-grade gliomas. The personalized treatment recommendation system by the machine learning model offers a promising approach to identify patients who are most likely to benefit from aggressive treatment strategies.

## Data Availability

The datasets presented in this study can be found in online repositories. The names of the repository/repositories and accession number(s) can be found below: The data and codes used for analysis in this study are available on GitHub at: https://github.com/NeuroCQ/EPHGG.

## Supplementary material

Supplementary Figure 1 Model performance of three nomogram models by time-dependent Area Under the Curve (tdAUC) in the external testing cohort.
