# Supplementary material for: Evaluating treatment strategies and machine learning based treatment recommendation system for elderly patients with high grade gliomas
Source: Front Oncol. 2025 Aug 11;15:1597925. doi: 10.3389/fonc.2025.1597925 (PMC12375445; doi:10.3389/fonc.2025.1597925)
Supplement: Supplementary file 1 [file Table1.docx]

|  | **SA** | **SAT** | **p-value** |
| --- | --- | --- | --- |
|  | ***N=1007*** | ***N=1864*** |  |
| Age: |  |  | <0.001 |
| ≥73 | 617 (61.3%) | 687 (36.9%) |  |
| 65-72 | 390 (38.7%) | 1177 (63.1%) |  |
| Gender: |  |  | <0.001 |
| Female | 516 (51.2%) | 815 (43.7%) |  |
| Male | 491 (48.8%) | 1049 (56.3%) |  |
| Marital: |  |  | <0.001 |
| Married | 599 (59.5%) | 1318 (70.7%) |  |
| UnMarried | 408 (40.5%) | 546 (29.3%) |  |
| Race: |  |  | 0.566 |
| Minority | 83 (8.24%) | 141 (7.56%) |  |
| White | 924 (91.8%) | 1723 (92.4%) |  |
| Type: |  |  | 0.037 |
| GBM | 782 (77.7%) | 1510 (81.0%) |  |
| nonGBM | 225 (22.3%) | 354 (19.0%) |  |
| Stage: |  |  | 0.116 |
| Advanced | 154 (15.3%) | 244 (13.1%) |  |
| Localized | 853 (84.7%) | 1620 (86.9%) |  |
| Grade: |  |  | 0.036 |
| III | 77 (7.65%) | 104 (5.58%) |  |
| IV | 930 (92.4%) | 1760 (94.4%) |  |
| TumorSize: |  |  | 0.209 |
| <=3cm | 245 (24.3%) | 495 (26.6%) |  |
| >3cm | 762 (75.7%) | 1369 (73.4%) |  |

**SupTable 1.** The Demographic and Clinicopathological Data of The Two Groups: Surgery Alone (SA) vs. Surgery with Adjuvant Therapy (SAT) before propensity score matching (PSM).

|  | **SA** | **SAT** | **p-value** |
| --- | --- | --- | --- |
|  | ***N=754*** | ***N=754*** |  |
| Age: |  |  | 0.877 |
| ≥73 | 366 (48.5%) | 362 (48.0%) |  |
| 65-72 | 388 (51.5%) | 392 (52.0%) |  |
| Gender: |  |  | <0.001 |
| Female | 383 (50.8%) | 281 (37.3%) |  |
| Male | 371 (49.2%) | 473 (62.7%) |  |
| Marital: |  |  | 1.000 |
| Married | 506 (67.1%) | 505 (67.0%) |  |
| UnMarried | 248 (32.9%) | 249 (33.0%) |  |
| Race: |  |  | 0.850 |
| Minority | 62 (8.22%) | 59 (7.82%) |  |
| White | 692 (91.8%) | 695 (92.2%) |  |
| Type: |  |  | 0.272 |
| GBM | 596 (79.0%) | 614 (81.4%) |  |
| nonGBM | 158 (21.0%) | 140 (18.6%) |  |
| Stage: |  |  | 0.823 |
| Advanced | 107 (14.2%) | 103 (13.7%) |  |
| Localized | 647 (85.8%) | 651 (86.3%) |  |
| Grade: |  |  | 0.118 |
| III | 50 (6.63%) | 35 (4.64%) |  |
| IV | 704 (93.4%) | 719 (95.4%) |  |
| TumorSize: |  |  | 0.672 |
| <=3cm | 184 (24.4%) | 176 (23.3%) |  |
| >3cm | 570 (75.6%) | 578 (76.7%) |  |

**SupTable 2.** The Demographic and Clinicopathological Data of The Two Groups: Surgery Alone (SA) vs. Surgery with Adjuvant Therapy (SAT) after propensity score matching (PSM).

|  | **AA** | **SAT** | **p-value** |
| --- | --- | --- | --- |
|  | ***N=499*** | ***N=1864*** |  |
| Age: |  |  | <0.001 |
| ≥73 | 228 (45.7%) | 687 (36.9%) |  |
| 65-72 | 271 (54.3%) | 1177 (63.1%) |  |
| Gender: |  |  | 0.901 |
| Female | 216 (43.3%) | 815 (43.7%) |  |
| Male | 283 (56.7%) | 1049 (56.3%) |  |
| Marital: |  |  | 0.893 |
| Married | 355 (71.1%) | 1318 (70.7%) |  |
| UnMarried | 144 (28.9%) | 546 (29.3%) |  |
| Race: |  |  | 0.406 |
| Minority | 44 (8.82%) | 141 (7.56%) |  |
| White | 455 (91.2%) | 1723 (92.4%) |  |
| Type: |  |  | <0.001 |
| GBM | 319 (63.9%) | 1510 (81.0%) |  |
| nonGBM | 180 (36.1%) | 354 (19.0%) |  |
| Stage: |  |  | <0.001 |
| Advanced | 150 (30.1%) | 244 (13.1%) |  |
| Localized | 349 (69.9%) | 1620 (86.9%) |  |
| Grade: |  |  | 0.928 |
| III | 29 (5.81%) | 104 (5.58%) |  |
| IV | 470 (94.2%) | 1760 (94.4%) |  |
| TumorSize: |  |  | 0.133 |
| <=3cm | 150 (30.1%) | 495 (26.6%) |  |
| >3cm | 349 (69.9%) | 1369 (73.4%) |  |

**SupTable 3.** The Demographic and Clinicopathological Data of The Two Groups: Adjuvant Therapy Alone (AA) vs. Surgery with Adjuvant Therapy (SAT) before propensity score matching (PSM).

|  | **AA** | **SAT** | **p-value** |
| --- | --- | --- | --- |
|  | ***N=369*** | ***N=369*** |  |
| Age: |  |  | 0.036 |
| ≥73 | 165 (44.7%) | 136 (36.9%) |  |
| 65-72 | 204 (55.3%) | 233 (63.1%) |  |
| Gender: |  |  | 0.001 |
| Female | 166 (45.0%) | 211 (57.2%) |  |
| Male | 203 (55.0%) | 158 (42.8%) |  |
| Marital: |  |  | 0.002 |
| Married | 261 (70.7%) | 219 (59.3%) |  |
| UnMarried | 108 (29.3%) | 150 (40.7%) |  |
| Race: |  |  | 0.772 |
| Minority | 24 (6.50%) | 27 (7.32%) |  |
| White | 345 (93.5%) | 342 (92.7%) |  |
| Type: |  |  | 1.000 |
| GBM | 268 (72.6%) | 269 (72.9%) |  |
| nonGBM | 101 (27.4%) | 100 (27.1%) |  |
| Stage: |  |  | 0.923 |
| Advanced | 65 (17.6%) | 67 (18.2%) |  |
| Localized | 304 (82.4%) | 302 (81.8%) |  |
| Grade: |  |  | 0.070 |
| III | 17 (4.61%) | 30 (8.13%) |  |
| IV | 352 (95.4%) | 339 (91.9%) |  |
| TumorSize: |  |  | 0.464 |
| <=3cm | 111 (30.1%) | 101 (27.4%) |  |
| >3cm | 258 (69.9%) | 268 (72.6%) |  |

**SupTable 4.** The Demographic and Clinicopathological Data of The Two Groups: Adjuvant Therapy (AA) vs. Surgery with Adjuvant Therapy (SAT) after propensity score matching (PSM).

|  | **STA** | **GTA** | **p-value** |
| --- | --- | --- | --- |
|  | ***N=1501*** | ***N=363*** |  |
| Age: |  |  | 0.043 |
| ≥73 | 536 (35.7%) | 151 (41.6%) |  |
| 65-72 | 965 (64.3%) | 212 (58.4%) |  |
| Gender: |  |  | 0.300 |
| Female | 647 (43.1%) | 168 (46.3%) |  |
| Male | 854 (56.9%) | 195 (53.7%) |  |
| Marital: |  |  | 0.506 |
| Married | 1067 (71.1%) | 251 (69.1%) |  |
| UnMarried | 434 (28.9%) | 112 (30.9%) |  |
| Race: |  |  | 0.652 |
| Minority | 111 (7.40%) | 30 (8.26%) |  |
| White | 1390 (92.6%) | 333 (91.7%) |  |
| Type: |  |  | 0.045 |
| GBM | 1202 (80.1%) | 308 (84.8%) |  |
| nonGBM | 299 (19.9%) | 55 (15.2%) |  |
| Stage: |  |  | 0.002 |
| Advanced | 215 (14.3%) | 29 (7.99%) |  |
| Localized | 1286 (85.7%) | 334 (92.0%) |  |
| Grade: |  |  | 0.751 |
| III | 82 (5.46%) | 22 (6.06%) |  |
| IV | 1419 (94.5%) | 341 (93.9%) |  |
| TumorSize: |  |  | 0.228 |
| <=3cm | 389 (25.9%) | 106 (29.2%) |  |
| >3cm | 1112 (74.1%) | 257 (70.8%) |  |

**SupTable 5.** Demographic and Clinicopathological Data of The Two Groups: Subtotal resection plus Adjuvant Therapy (STA) Vs. Gross total resection plus Adjuvant Therapy (GTA) before propensity score matching (PSM).

|  | **STA** | **GTA** | **p-value** |
| --- | --- | --- | --- |
|  | ***N=363*** | ***N=363*** |  |
| Age: |  |  | 1.000 |
| ≥73 | 151 (41.6%) | 151 (41.6%) |  |
| 65-72 | 212 (58.4%) | 212 (58.4%) |  |
| Gender: |  |  | 1.000 |
| Female | 169 (46.6%) | 168 (46.3%) |  |
| Male | 194 (53.4%) | 195 (53.7%) |  |
| Marital: |  |  | 0.936 |
| Married | 249 (68.6%) | 251 (69.1%) |  |
| UnMarried | 114 (31.4%) | 112 (30.9%) |  |
| Race: |  |  | 0.891 |
| Minority | 28 (7.71%) | 30 (8.26%) |  |
| White | 335 (92.3%) | 333 (91.7%) |  |
| Type: |  |  | 0.917 |
| GBM | 310 (85.4%) | 308 (84.8%) |  |
| nonGBM | 53 (14.6%) | 55 (15.2%) |  |
| Stage: |  |  | 1.000 |
| Advanced | 29 (7.99%) | 29 (7.99%) |  |
| Localized | 334 (92.0%) | 334 (92.0%) |  |
| Grade: |  |  | 1.000 |
| III | 21 (5.79%) | 22 (6.06%) |  |
| IV | 342 (94.2%) | 341 (93.9%) |  |
| TumorSize: |  |  | 0.805 |
| <=3cm | 102 (28.1%) | 106 (29.2%) |  |
| >3cm | 261 (71.9%) | 257 (70.8%) |  |

**SupTable 6.** Demographic and Clinicopathological Data of The Two Groups: Subtotal resection plus Adjuvant Therapy (STA) Vs. Gross total resection plus Adjuvant Therapy (GTA) after propensity score matching (PSM).
