# Supplementary figures and images for: Evaluating treatment strategies and machine learning based treatment recommendation system for elderly patients with high grade gliomas
Source: Front Oncol. 2025 Aug 11;15:1597925. doi: 10.3389/fonc.2025.1597925 (PMC12375445; doi:10.3389/fonc.2025.1597925)

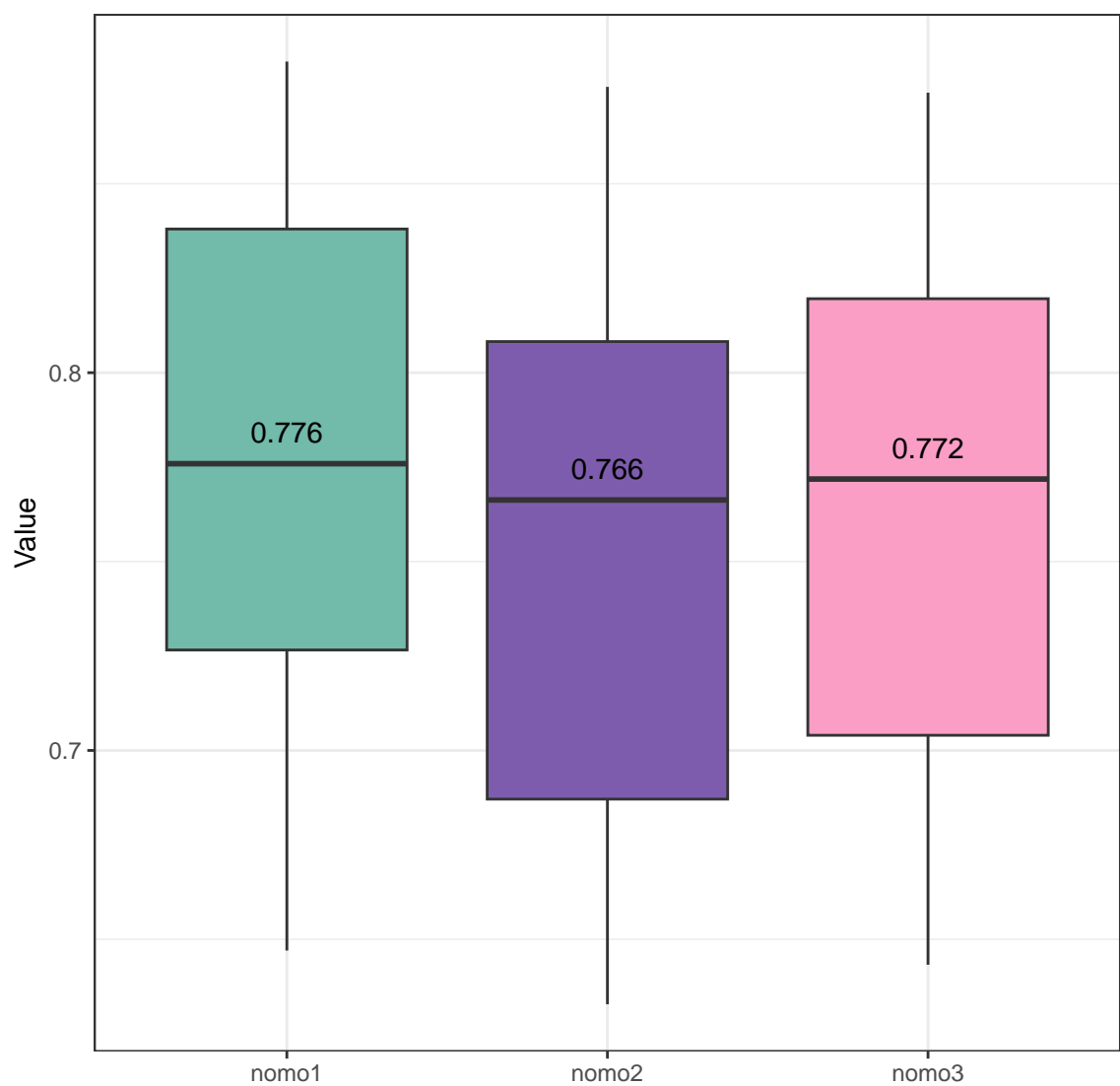

Supplement: Supplementary Figure 1 — Model performance of three nomogram models by time-dependent Area Under the Curve (tdAUC) in the external testing cohort. [file DataSheet1.pdf]
